# Evaluation of the Immunogenicity in Mice Orally Immunized with Recombinant *Lactobacillus casei* Expressing Porcine Epidemic Diarrhea Virus S1 Protein

**DOI:** 10.3390/v14050890

**Published:** 2022-04-25

**Authors:** Ya Xiao, Xiaona Wang, Yue Li, Fengsai Li, Haiyuan Zhao, Yilan Shao, Liu Zhang, Guojie Ding, Jiaxuan Li, Yanping Jiang, Wen Cui, Zhifu Shan, Han Zhou, Li Wang, Xinyuan Qiao, Lijie Tang, Yijing Li

**Affiliations:** 1 College of Veterinary Medicine, Northeast Agricultural University, Harbin 150030, China; xiaoya19940616@126.com (Y.X.); xiaonawang0319@163.com (X.W.); y1925729473@163.com (Y.L.); yilvwenrou@126.com (F.L.); shaoyilan232@163.com (Y.S.); zl18800430475@163.com (L.Z.); lijiaxuan.1993@163.com (J.L.); jiangyanping2017@126.com (Y.J.); cuiwen_200@163.com (W.C.); shanzhifu@126.com (Z.S.); zhouhan9659@163.com (H.Z.); wanglicau@163.com (L.W.); qiaoxinyuan@126.com (X.Q.); tanglijie@neau.edu.cn (L.T.); 2 Heilongjiang Key Laboratory for Animal Disease Control and Pharmaceutical Development, Harbin 150030, China; 3 Jiangsu Hanswine Food Co., Ltd., Ma’anshan 151700, China; zhywxn1925@163.com; 4 Harbin Vikeses Biological Technology Co., Ltd., Harbin 150030, China; dingguojie1974@126.com

**Keywords:** PEDV S1 glycoprotein, recombinant *Lactobacillus*, mucosal immunity, oral vaccine

## Abstract

Porcine epidemic diarrhea (PED), characterized by diarrhea, vomiting, and dehydration, is an acute enteric infectious disease of pigs. The disease is caused by porcine epidemic diarrhea virus (PEDV), which infects the intestinal mucosal surface. Therefore, mucosal immunization through the oral route is an effective method of immunization. Lactic acid bacteria, which are acid resistant and bile-salt resistant and improve mucosal immunity, are ideal carriers for oral vaccines. The S1 glycoprotein of PEDV mediates binding of the virus with cell receptors and induces neutralizing antibodies against the virus. Therefore, we reversely screened the recombinant strain pPG-SD-S1/Δ*upp ATCC 393* expressing PEDV S1 glycoprotein by *Lactobacillus casei* deficient in *upp* genotype (Δ*upp ATCC 393*). Mice were orally immunized three times with the recombinant bacteria that had been identified for expression, and the changes of anti-PEDV IgG and secreted immunoglobulin A levels were observed over 70 days. The results indicated that the antibody levels notably increased after oral administration of recombinant bacteria. The detection of extracellular cytokines on the 42nd day after immunization indicated high levels of humoral and cellular immune responses in mice. The above results demonstrate that pPG-SD-S1/Δ*upp ATCC 393* has great potential as an oral vaccine against PEDV.

## 1. Introduction

Porcine epidemic diarrhea (PED) is an acute viral disease of pigs that causes huge economic losses to the global agriculture industry [1,2]. Presently, the RNA vaccine and inactivated whole virus vaccine developed by Harrisvaccines^TM^ and Zoetis, respectively, are widely used and effective against PEDV [3]. Porcine epidemic diarrhea virus (PEDV), which mainly causes intestinal epithelial cell damage in neonatal pigs, is the etiological agent of PED; therefore, oral vaccines that effectively stimulate the intestinal mucosal immune response have proven valuable for practical application [4]. To achieve an adequate level of mucosal immune response at the relevant site, oral vaccines must be protected against a harsh digestive environment [5]. Therefore, effective antigen delivery vehicles are crucial for oral vaccines.

Lactic acid bacteria (LAB) have been reported to effectively induce mucosal immune response as an oral vaccine vector for enteroviruses [6,7]. *Lactobacillus casei*, a type of LAB, has many beneficial properties that make it an ideal carrier for antigen presentation. *L. casei* can survive, colonize, and exert intrinsic adjuvant activity in the upper gastrointestinal tract [8]. Furthermore, *L. casei* can effectively induce the production of secreted immunoglobulin A (SIgA) and enhance both humoral and cellular immunity [9,10,11]. The *upp* gene, encoding uracil phosphoribosyltransferase (UPRTase), which is involved in the purine and pyrimidine salvage pathways, is a widely used counter-selection marker in bacteria. UPRTase can convert 5-fluorouracil (5-FU) to 5-fluoro-UMP, inhibiting thymidylate synthase and causing cell death [12]. Compared with Δ*upp* mutant strains, upp-expressing bacteria are sensitive to 5-FU [13]. Furthermore, *upp* was not found to be essential in the genome analysis of *L. casei* [14]. Therefore, we constructed *upp* gene-deleted *L. casei ATCC 393* (Δ*upp ATCC 393*), providing a new screening method for obtaining recombinant bacterial strains.

The genome of PEDV is 28.5 kb in size and contains at least seven open reading frames (ORF), which code for the spike (S), envelope (E), membrane (M), nucleocapsid (N), ORF1a, ORF1b, and ORF3 proteins [15,16]. The S protein, which is the principal antigenic determinant, is closely associated with virus–host recognition and neutralizing antibodies produced and can be divided into S1 and S2 proteins by cleavage at a specific site [2,17]. The S1 protein, an important determinant of virulence, contributes to receptor recognition of neutralizing epitopes [18,19,20]. Therefore, it is the main target gene for vaccine development.

The predominant antibody isotype on mucosal surfaces, sIgA, can prevent bacterial and viral infections by establishing the defense of the intestinal mucosa [21,22]. The SIgA antibody found in colostrum is an excellent source for piglets to obtain passive immune protection, illustrating its importance in controlling PEDV infection [4]. Therefore, we constructed a recombinant LAB expressing *Lactobacillus* Ribosome Binding Site (SD) and the PEDV S1 glycoprotein for oral immunization to increase the level of sIgA in the intestinal mucosa.

In this study, a recombinant Δ*upp L. casei* strain expressing the PEDV S1 protein was developed and evaluated for its potency as an oral vaccine. Changes in SIgA and IgG were monitored during the 70-day period after immunization, which provided the basis for the preparation of an effective oral PEDV vaccine.

## 2. Materials and Methods

### 2.1. Virus, Plasmid and Bacterial Strain

Δ*upp L. casei ATCC 393* (Δ*upp ATCC 393*) was grown in de Man, Rogosa, and Sharpe (MRS) broth at 37 °C in a stationary state. The PEDV LJB/15 strain, isolated and identified in our laboratory, was propagated in Vero-L cells at 37 °C and 5% CO_2_. PEDV LJB2019, the parental sequence of the S1 glycoprotein gene in this experiment, was a PEDV epidemic strain amplified from clinical samples collected in a diseased pig farm in Heilongjiang Province, China in 2019. The *Escherichia coli*–LAB shuttle vector pPG-T7g10-PPT was constructed in our laboratory.

### 2.2. Construction of pPG-SD-S1/Δupp ATCC 393

**Figure 1:**
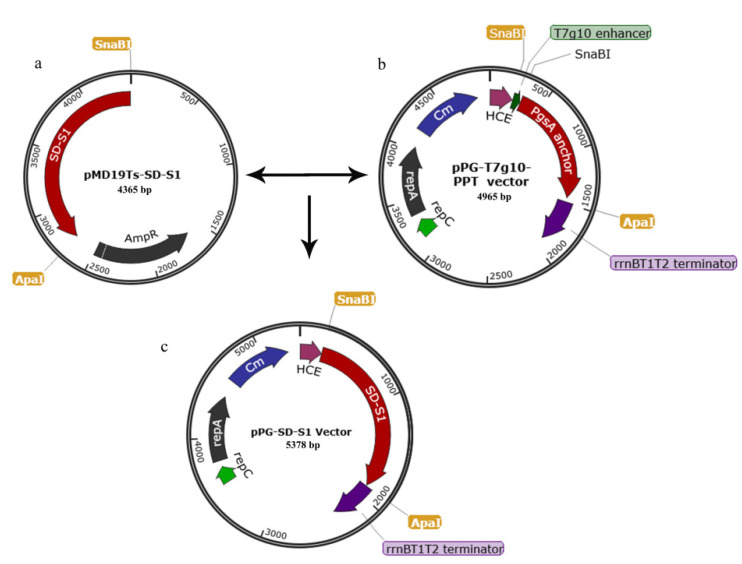
Schematic diagram of recombinant plasmid construction. Constitutive cell surface expression plasmid pPG-T7g10-PPT (**a**), cloning vector pMD19Ts-SD-S1 (**b**) and recombinant plasmid pPG-SD-S1 (**c**). Fusion DNA fragment SD-S1 (Lactobacillus Ribosome Binding Site sequence (SD) and PEDV S1 glycoprotein gene) obtained from pMD19Ts-SD-S1 by SnaB I and Apa I digestion was inserted into the corresponding sites of plasmid pPG-T7g10-PPT, generating recombinant plasmid pPG-SD-S1.

The construction method for the recombinant plasmid is outlined in Figure 1. After the extraction of PEDV genomic RNA from PEDV LJB2019, the PEDV S1 gene was subjected to a reverse transcription (RT)-polymerase chain reaction (PCR). The *Lactobacillus* Ribosome Binding Site sequence (SD) was connected to the 5′ end of the S1 gene (The S1 gene sequence is shown in Figure S1.) by fusion PCR (Fusion PCR primer sequences are shown in Table 1). The SD-S1 (Figure 1a) fragment was linked to the plasmid pPG-T7g10-PPT (Figure 1b) by restriction enzyme digestion, generating the plasmid pPG-SD-S1. The recombinant plasmid pPG-SD-S1 (Figure 1c) was transformed into Δ*upp ATCC 393* competent cells by electroporation [23], generating the recombinant strain pPG-SD-S1*/*Δ*upp ATCC 393*.

### 2.3. Protein Expression

The strain pPG-SD-S1*/*Δ*upp ATCC 393* was inoculated into MRS broth (1:100) and incubated for 12 h at 37 °C. Next, the culture was centrifuged at 4 °C, and the pellet and supernatant obtained were sonicated. Further, proteins in the sonicated supernatant and pellet were separated using 10% sodium dodecyl sulfate-polyacrylamide gel electrophoresis and were electrotransferred onto polyvinylidene fluoride membranes (Millipore, Milford, MA, USA). The membranes were incubated with mouse S1 monoclonal antibody (stored in our laboratory) as the primary antibody for 1 h at 37 °C and horseradish peroxidase (HRP)-conjugated goat anti-mouse IgG antibody (1:5000) (Thermo Scientific, Durham, NC, USA) as the secondary antibody for 1 h at 37 °C. The results were observed using a chemiluminescent substrate reagent (Solarbio, Beijing, China) according to the manufacturer’s instructions.

### 2.4. Immunization and Sample Collection

**Figure 2:**
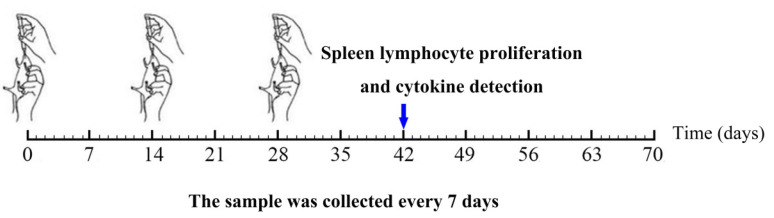
The timeline of mice immunization procedure and sample collection. Mice (*n* = 90) were equally divided into three groups. The black font represents the days of immunization; a sample was collected every seven days. Spleen lymphocyte proliferation assay and cytokine detection were performed on day 42.

To evaluate the immunogenicity of pPG-SD-S1*/*Δ*upp L. casei 393* as an oral vaccine, 35-day-old female specific pathogen-free (SPF) BALB/c mice (*n* = 90) were housed in an SPF environment and provided with adequate water and food for the standard. The recombinant strains were inoculated in MRS broth (1:100) and cultured for 14 h at 37 °C. Further, the cultures were washed and diluted to a final concentration of 10^10^ colonu-forming units (CFU)/mL with phosphate-buffered saline (PBS). Three groups of mice were administered 200 µL PBS, Δ*upp ATCC 393,* or pPG-SD-S1*/*Δ*upp ATCC 393* (30 mice per group). As shown in Figure 2, each mouse was immunized three times with an immunization cycle of 3 days, and each immunization was 14 days apart. To detect IgG and SIgA, the sera, tears, nasal fluid, genital mucus, intestinal mucus, and feces of the immunized mice were collected at 0, 7, 14, 21, 28, 35, 42, 49, 56, 63, and 70 days after the first immunization and stored at −40 °C until use. Of these, the intestinal mucus and feces required pretreatment. Intestinal mucus was flushed from the intestine using HEPES buffer. After incubation and centrifugation, the supernatant was stored at −40 °C until further use. In addition, 400 µL of 1% bovine serum albumin (BSA) and 1 mmol/L phenylmethylsulfonyl fluoride (Sigma, Ronkonkoma, NY, USA) were added to 0.1 g of feces, processed, and saved as previously described [24].

### 2.5. Enzyme-Linked Immunosorbent Assay (ELISA)

Changes in anti-PEDV IgG in serum were detected by indirect ELISA. The same method was used to monitor the levels of the SIgA antibody. Briefly, after overnight storage at 4 °C, PEDV was coated with polystyrene microtiter plates. The plates were washed with 100 µL PBS-0.1% Tween 20, 5% skim milk was added to each well, and the plate was incubated for 2 h at 37 °C. After the 100 µL samples were added (each sample was added in triplicates), the plate was incubated for 2 h at 37 °C. Further, 100 µL HRP-conjugated goat anti-mouse IgG/IgA antibody (1:5000) (Thermo Scientific, Durham, NC, USA) was added to the plate for 1 h at 37 °C. Finally, o-phenylenediamine dihydrochloride (Sigma, Ronkonkoma, NY, USA) was added as substrate, and absorbance was recorded at 490 nm.

### 2.6. Detection of PEDV Neutralizing Antibody Activity in Serum

To determine the neutralizing activity of anti-PEDV IgG in serum, 50 µL of serum from each immunized mouse was collected on the 42nd day after immunization and serially diluted (two-fold). The mixture of diluted serum and 50 µL of 50% tissue culture infected (TCID_50_) PEDV was plated at 37 °C, incubated for 1 h, and placed on a Vero-L cell monolayer in a 96-well plate at 37 °C for 1 h. The culture medium was replenished after discarding the solution. The presence of a PEDV-specific cytopathic effect was observed after two days incubation at 37 °C and 5% CO_2_. In this study, eight biological replicates and three technical replicates were set for each sample. In addition, a negative serum, positive serum, blank, and virus control were included in each experiment.

### 2.7. Lymphocyte Proliferation and Cytokine Detection

Three mice from each group were sacrificed on the 42nd day, and spleen cells were obtained under sterile conditions for the detection of spleen lymphocyte proliferation. Briefly, splenocytes at a concentration of 5 × 10^6^ cells/mL (three replicates) were cultured in 96-well plates with RPMI1640 + 20% fetal bovine serum at 37 °C and 5% CO_2_. Splenocytes were stimulated with purified PEDV S1 protein at 1, 5, or 25 µg/mL for 60 h at 37 °C with 5% CO_2_. Simultaneously, 5 µg/mL concanavalin A (Con A) was set as a positive control, and RPMI1640 medium was set as a negative control. Spleen lymphocytes proliferation, detected by the CellTiter 96^®^ AQueous Non-Radioactive Cell Proliferation Assay (Promega, Madison, WI, USA), was evaluated at 570 nm absorbance, according to the manufacturer’s instructions. Bars represented mean *±* standard error of each group. According to the manufacturer’s instructions (Biosource International Inc., Camarillo, CA, USA), serum interleukin-2 (IL-2), interferon-γ (IFN-γ), IL-4, IL-12, IL-10, and IL-17 levels were detected using antigen capture ELISA. All of the above experiments were repeated three times, and the cytokine concentration was calculated based on the standard curve.

### 2.8. Statistical Analysis

The data are the mean of three replicates for a single sample ± standard error. GraphPad Prism v 5.0 (San Diego, CA, USA) was used for the statistical analysis of the data. Tukey’s multiple comparison test and two-way analysis of variance (ANOVA) were used to analyze the significance of differences between the means. Differences with *p* values less than 0.05 (*p* < 0.05) and less than 0.01 (*p* < 0.01) were considered significant and highly significant, respectively.

## 3. Results

### 3.1. Protein Expression

**Figure 3:**
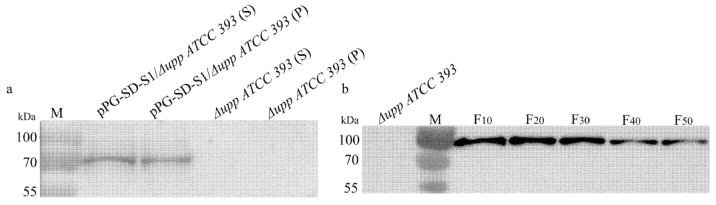
The expression and stability of the target protein was identified in Western blots using a mouse anti-S1 monoclonal antibody (**a**,**b**). pPG-SD-S1*/*Δ*upp ATCC 393* lysate supernatant [pPG-SD-S1*/*Δ*upp ATCC393* (S)] and pellet [pPG-SD-S1*/*Δ*upp ATCC393* (P)] show relevant immunoreactive bands, but the supernatant (Δ*upp ATCC393* (S)) and pellet (Δ*upp ATCC393* (P)) of Δ*upp ATCC 393* lysate do not. The relevant immunoreactive bands are evident in the pPG-SD-S1*/*Δ*upp ATCC 393* lysate from the 10th to 50th generations, but not in Δ*upp ATCC 393*. M: protein molecular weight marker.

pPG-SD-S1*/*Δ*upp ATCC 393* and Δ*upp ATCC 393* were cultured overnight, centrifuged, and lysed for Western blotting. The bands of predictable size appeared in the supernatant and pellet of the pPG-SD-S1*/*Δ*upp ATCC 393* lysate but not in the supernatant and pellet of the Δ*upp ATCC 393* lysate, indicating that the target protein was effectively expressed (Figure 3a). To confirm that the recombinant bacteria stably expressed the target protein, overnight cultures from the 10th, 20th, 30th, 40th, and 50th generations of pPG-SD-S1*/*Δ*upp ATCC 393* were collected, centrifuged, and lysed for Western blotting. Predictable bands appeared in the 10th to 50th generation of pPG-SD-S1*/*Δ*upp ATCC 393* lysates (Figure 3b), whereas bands were not visible in the Δ*upp ATCC 393* lysates. The above results indicate that the recombinant bacteria stably expressed the target protein.

### 3.2. Changes in IgG Levels Induced by Oral Immunization

**Figure 4:**
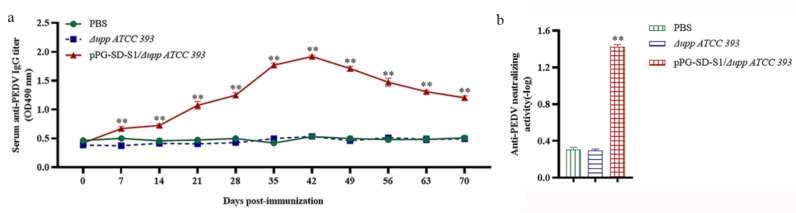
Determination of anti-porcine epidemic diarrhea virus (PEDV) specific IgG antibody (**a**) and anti-PEDV neutralizing activity in mice post-immunization (**b**). The levels of anti-PEDV IgG antibody were measured in the sera of immunized mice using indirect ELISA. The polyline represents changes in the anti-PEDV IgG level in orally immunized mice. Anti-PEDV neutralizing antibodies were detected by plaque reduction assay performed with dilutions of serum samples taken at the 42nd day post-immunization. Bars represent the mean ± standard error in each group (** *p* < 0.01 compared to the control groups: PBS and Δ*upp ATCC 393*).

Changes in anti-PEDV IgG antibody levels induced by pPG-SD-S1*/*Δ*upp ATCC 393* were detected by ELISA. As shown in Figure 4a, anti-PEDV IgG levels started to increase on day 7 and peaked on day 42 of the oral immunization with recombinant bacteria. In contrast, anti-PEDV IgG levels in the PBS and Δ*upp ATCC 393* groups did not change significantly (*p* < 0.05). In addition, the sera from immunized mice exhibited anti-PEDV neutralizing activity. The anti-PEDV neutralizing antibodies (IgG) in the serum obtained from mice orally immunized with pPG-SD-S1/Δ*upp ATCC 393* (1:28) were significantly higher than that with oral PBS (1:2) and Δ*upp ATCC 393* (1:2) (Figure 4b). The above results show that oral immunization with recombinant bacteria can effectively induce high levels of anti-PEDV immune response in mice.

### 3.3. Changes in SIgA Levels Induced by Oral Immunization

**Figure 5:**
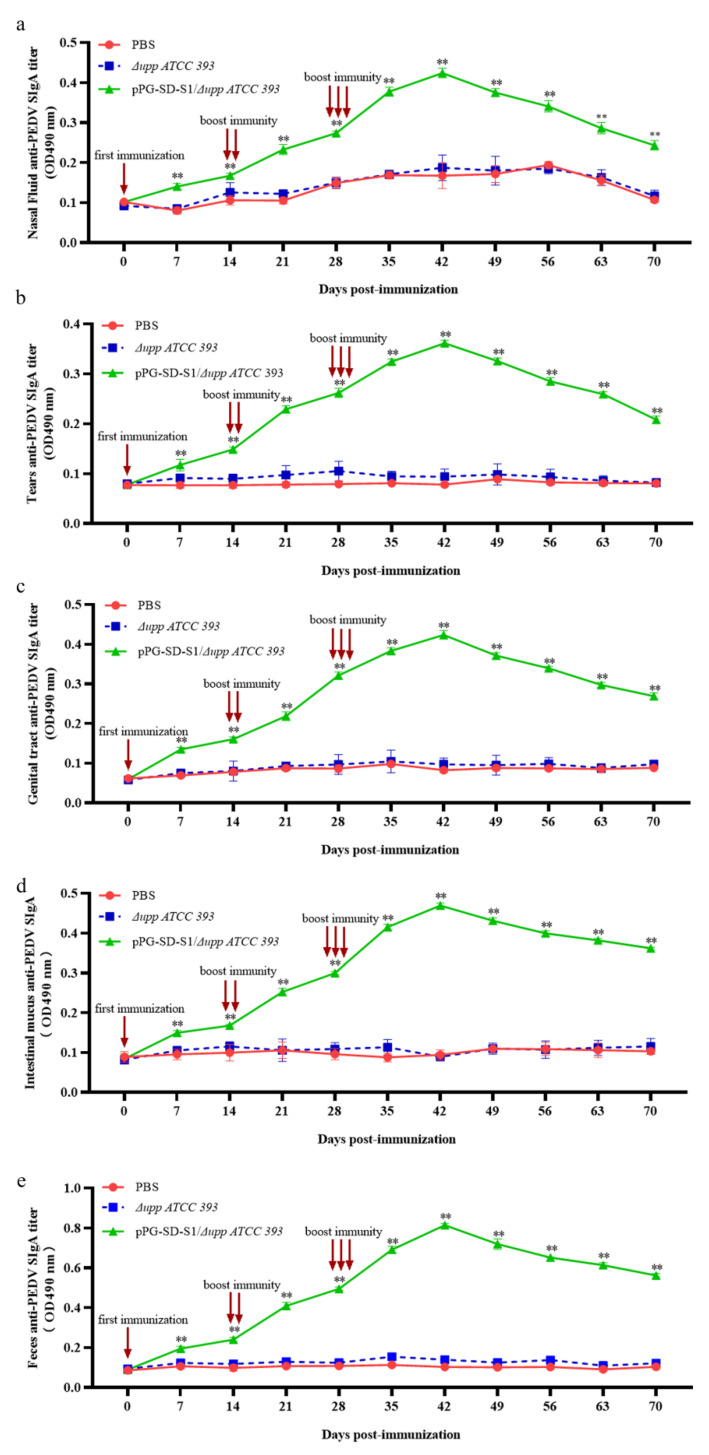
The changes in anti-PEDV-specific SIgA antibody levels in the nasal fluid (**a**); tears (**b**); reproductive tract mucus (**c**); intestinal mucus (**d**); and feces (**e**) of immunized mice. The intestinal mucus was gently scraped from the excised intestinal tissue with HEPES buffer, and 0.1 g of feces was added to 400 µL of 1 mmol/L phenylmethylsulfonyl fluoride and 1% BSA. After incubation and centrifugation, the supernatant was stored at −40 °C until use. The anti-PEDV SIgA levels were measured using indirect ELISA. (** *p* < 0.01 compared to the control groups: PBS and Δ*upp ATCC 393*).

To evaluate the mucosal immune response induced by oral recombinant bacteria in mice, ELISA was used to detect anti-PEDV SIgA levels in the tears, nasal fluid, genital mucus, intestinal mucus, and fecal samples. The levels of SIgA in the nasal fluid (Figure 5a), tears (Figure 5b), genital mucus (Figure 5c), intestinal mucus (Figure 5d), and feces (Figure 5e) in the oral pPG-SD-S1/Δ*upp ATCC 393* immunized group were significantly higher than those in the oral PBS and Δ*upp ATCC 393* groups and increased significantly on day 7 and peaked at 42 days. In contrast, anti-PEDV SIgA in the oral PBS and Δ*upp ATCC 393* groups did not show a significant change. The above results indicate that pPG-SD-S1/Δ*upp ATCC 393* could effectively induce a mucosal immune response in mice.

### 3.4. Detection of Cytokines in Serum

**Figure 6:**
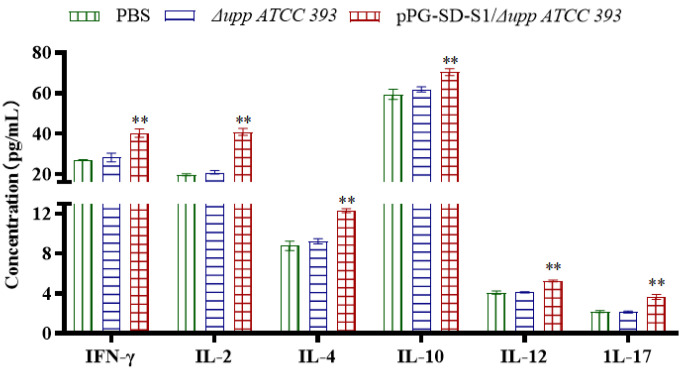
Determination of cytokine levels in immunized mice. Cytokine levels, including IFN-γ, IL-2, IL-4, IL-10, IL-12 and IL-17, were detected in the sera of mice on the 42nd day after the primary immunization. The concentrations of cytokines were calculated according to the standard curve. Bars represent mean ± standard error in each group (** *p* < 0.01 compared to the control groups: PBS and Δ*upp ATCC 393*).

To determine the type of immune response induced by oral recombinant bacteria, changes in cytokine levels were detected on the 42nd day. Compared to the oral PBS and Δ*upp ATCC 393* groups, the levels of cytokines IL-2, IFN-γ, IL-4, IL-12, IL-10, and IL-17 in the sera of the oral pPG-SD-S1/Δ*upp ATCC 393* group were significantly increased (Figure 6). These results show that oral administration of pPG-SD-S1/Δ*upp ATCC 393* could significantly stimulate the generation of Th1, Th2, and Th17 cellular immunity in mice.

### 3.5. Lymphocyte Proliferation

**Figure 7:**
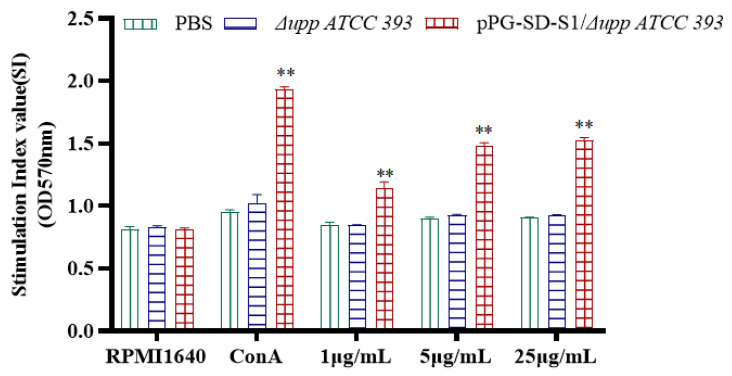
Lymphocyte proliferation in immunized mice was determined by the 3-(4,5-dimethylthiazol-2-yl)-2,5-diphenyltetrazolium bromide (MTT) assay. With purified PEDV S1 protein as the stimulation source, the stimulation index of spleen lymphocytes isolated from immunized mice was detected by the MTT assay. Bars represent mean ± standard error in each group (** *p* < 0.01, compared to controls: PBS and Δ*upp ATCC 393*).

Using Con A as a positive control and RPMI1640 as a negative control, isolated splenocytes were restimulated in vitro with PEDV S1 pure protein, followed by 3-(4,5-dimethylthiazol-2-yl)-2,5- diphenyltetrazolium bromide (MTT) assay. Compared with the oral PBS and Δ*upp ATCC 393* groups, the stimulation index of the oral pPG-SD-S1/Δ*upp ATCC 393* immunization group was significantly increased and showed a dose-dependent phenomenon (Figure 7).

## 4. Discussion

Currently, PED has become one of the major infectious diseases in pig farms, leading to heavy economic losses to the farming industry. The main surface antigen of PEDV is the spike protein (S), which is present on the capsular membrane [25]. The S protein interacts with specific host cell receptors, mediating the fusion of the virus with the cell membrane and permitting the virus to enter susceptible cells [26]; therefore, the protein is crucial for viral infection. Experimental results prove that neutralizing antibodies, which are closely associated with immune protection against viruses, could be induced by the S protein [27]. Based on the classic PEDV CV777 strain, the S protein is divided into the S1 (AA 1–726) and S2 (AA 727–1386) functional regions [28]. The plants such as tobacco or rice callus have been used for expressing PEDV S1 protein, and significant humoral and mucosal immune responses were induced in animals after feeding on them [29,30]. Therefore, in this study, the S1 glycoprotein gene amplified from the PEDV epidemic strain, with over 97% similarity with PEDV strains prevalent in recent years, was selected as the target gene. The strain contains the neutralizing antibody epitope and is a good candidate antigen for vaccines [31].

Presently, most vaccines against PEDV are intramuscular inactivated vaccines [32]. As PEDV infections mainly occur on the intestinal mucosal epithelial surface, mucosal immunity plays a crucial role in preventing PEDV infections [33]. Oral immunization effectively stimulates the digestive tract to produce local mucosal immunity, causing a systemic immune response [34]. LAB, having advantages such as acid resistance, bile salt resistance, avoidance of immune tolerance, and mucosal immunity improvement, are extensively used as oral vaccine carriers [35]. Research has shown that *L. casei ATCC 393*, expressing a fusion protein of the PCV2 capsid protein and *E. coli* thermolabile toxin B subunit, stimulates a strong mucosal immune response against PCV2 in mice after oral immunization [36]. In addition, *L. plantarum*, which expresses severe acute respiratory syndrome coronavirus 2, can generate an immune response in the respiratory tract. These results suggest that LAB have the potential to become mucosal vaccines against COVID-19 [37]. Therefore, LAB is an ideal carrier for oral vaccines.

In this study, the SD sequence, the RBS and signal peptide sequence of *Lactobacillus casei* to improve protein expression, and the PEDV S1 glycoprotein were expressed by the shuttle vector *E. coli*–LAB in Δ*upp ATCC 393*. The recombinant strain was used for oral immunization to evaluate its immune efficacy in mice. In view of the highly mutant form of the PEDV S protein, the S1 gene selected in this experiment was amplified from the PEDV epidemic strains. Therefore, recombinant bacteria have practical applications, and the results of oral immunization in mice corroborate this fact. The experimental results showed that the oral administration of pPG-SD-S1/Δ*upp ATCC 393* could stimulate an increase in anti-PEDV IgG and SIgA levels in mice, indicating that pPG-SD-S1/Δ*upp ATCC 393* has the potential to act as a PEDV oral vaccine.

The gut is the primary site of PEDV infection and transmission; therefore, high levels of IgG and SIgA are required for an effective mucosal immune response against PEDV [38]. In addition, SIgA is also one of the evaluation criteria for the degree of virus infection and protective efficacy of vaccines [39,40]. The experimental results showed that anti-PEDV IgG antibodies increased after the oral administration of pPG-SD-S1/Δ*upp ATCC 393*, and this increase was more notable after booster immunization. Furthermore, antibody neutralization experiments demonstrated that IgG on day 42 after immunization (anti-PEDV-specific IgG antibody levels peak on this day) could complement immune defense by reducing the aggressiveness of PEDV. IgA has been shown to peak at six weeks in piglets and decrease at eight weeks [4]. Neonatal pigs could only be protected after 35 days of durable immunity. Therefore, changes in SIgA levels were monitored at 70 days after the primary immunization. The oral administration of pPG-SD-S1/Δ*upp ATCC 393* effectively induced the production of high levels of SIgA in the nasal fluid, tears, genital mucus, intestinal mucus, and feces of mice. Meanwhile, the levels of SIgA peaked at 42 days after the primary immunization, indicating that oral administration of pPG-SD-S1/Δ*upp ATCC 393* could effectively elicit the mucosal immune response.

Additionally, the secretion levels of cytokines IL-2, IFN-γ, IL-10, IL-4, IL-12, and IL-17 were significantly increased after oral immunization with pPG-SD-S1/Δ*upp ATCC 393*, indicating that the recombinant bacteria significantly stimulated the production of Th1, Th2, and Th17 cellular immunity in mice. Th1 responses are connected to cell-mediated immunity, whereas Th2 responses are related to humoral immunity [41]. IFN-γ and IL-2, which are Th1-type cytokines, assist antibody production, participate in cellular immune response, T cell proliferation, and induce cytotoxic effects [42,43]. IL-12 stimulates differentiation of T cells into Th1 or committed Th1 cells [44]. IL-4 stimulates the growth and differentiation of B lymphocytes, as well as antibody production. Moreover, IL-4 can also stimulate dendritic cells to produce IFN-γ, displaying a synergistic effect with IL-12 [44]. Both Th1 and Th2 cells can induce the production of IL-10 [43]. IL-17, which is produced by Th17 cells, is involved in neutrophil excitation, further stimulating the production of pro-inflammatory responses [45]. In this study, the cytokines level in serum was significantly increased on the 14th day after the third round of immunization, but oral administration of Δ*upp ATCC 393* could not produce a significant difference with the oral PBS group, indicating that oral administration of pPG-SD-S1/Δ*upp ATCC 393* can induce strong humoral and cellular immunity in mice simultaneously. Similar results were observed in spleen lymphocyte proliferation experiments.

In conclusion, oral immunization of pPG-SD-S1/Δ*upp ATCC 393* could stimulate mice to produce specific immune responses against PEDV. This study can be used as a reference to develop PEDV vaccines for piglets. In the future, we will insert the SD-S1 sequence into the genome of Lactobacillus casei, using the *upp* gene deficiency as a counter-selection marker, by homologous recombination. Future experiments will focus on the immune protection effect of the recombinant strains in a porcine model.

## 5. Conclusions

In conclusion, we developed an anti-PEDV vaccine for oral administration using recombinant *L. casei* to deliver the S1 antigen and demonstrated that the recombinant bacteria pPG-SD-S1/Δ*upp ATCC 393*, reverse screened by Δ*upp ATCC 393*, effectively induces an immune response against PEDV; therefore, this recombinant strain can be a potential oral PEDV vaccine.

## Figures and Tables

**Table 1:** Details of primers used in this study.

Target	ID	Primer Sequence (5′-3′)	PCR Size
SD+ Flag	SDF	**TACGTA**GCGAGGAGTGACGATAAAGATGAAATTAAAGCAA	161 bp
	SDR	CTTATCGTCGTCATCCTTGTAATCAAGTCGACCATCAGCTTTAACTGTTG	
S1	S1F	GTCGACTTGATTACAAGGATGACGACGATAAGTGCATTGGTTAT	1518 bp
	S1R	**GGGCCC**CTAGTAAAAGAAACCAGGCAACTC	

Bold type indicates restriction enzyme recognition sites used for cloning.

## Data Availability

Publicly available datasets were analyzed in this study. This data can be found in Supplementary Materials.

## Supplementary Materials

The following supporting information can be downloaded at: https://www.mdpi.com/article/10.3390/v14050890/s1, Figure S1: The S1 gene sequence used in this study.
