# Supplementary material for: Evaluation of the Immunogenicity in Mice Orally Immunized with Recombinant Lactobacillus casei Expressing Porcine Epidemic Diarrhea Virus S1 Protein
Source: Viruses. 2022 Apr 25;14(5):890. doi: 10.3390/v14050890 (PMC9145290; doi:10.3390/v14050890)
Supplement: Supplementary file 1 [file viruses-14-00890-s001.zip › viruses-1673173-supplementary.pdf]

Figure S1. The S1 gene sequence used in this study is as follows:

TGCATTGGTTATGCTGCCAATGTATTTGCTACTGAGCCCAATGGCCACATACCAGAAGG
TTTTAGTTTTAATAATTGGTTTCTTTTGTCCAATGATTCCACTTTGGTGCATGGTAAGGTG
GTTTCCAACCAACCATTGTTGGTCAATTGTCTTTTGGCCATGCCTAAGATTTATGGACTA
GGCCAATTTTTCTCCTTCAATCAAACGATCGATGGTGTTTGTAATGGAGCTGCTGTGCA
GCGTGCACCAGAGGCTCTGAGGTTTAATATTAATGACACCTCTGTCATTCTTGCTGAAG
GCTCAATTGTACTTCACACTGCTTTAGGAACAAATCTTTCTTTTGTTTGCAGTAATTCTT
CAGATCCTCATTTAGCTACCTTCACCATACCTCTGGGTGCTACCCAAGTACCCTATTATT
GTTTTCTTAAAGTGGATACTTACAACTCCACTGTTTATAAATTTTTGGCTGTTTTACCTCC
TACCGTCAGGGAAATTGTCATCACCAAGTATGGTGATGTTTATGTCAATGGGTTTGGAT
ACTTGCATCTCGGTTTGTTGGATGCTGTCACAATTAATTTCACTGGTCATGGCACTGAC
GATGATGTTTCTGGTTTTTGGACCATAGCATCGACTAATTTTGTTGATGCACTCATCGAA
GTTCAAGGAACTGCCATTCAGCGTATTCTTTATTGTGATGATCCTGTTAGCCAACTCAA
GTGTTCTCAGGTTGCTTTTGACCTTGACGATGGTTTTTACCCTATTTCTTCTAGAAACCT
TCTGAGTCATGAACAGCCAATTTCTTTTGTTACTCCGCCATCATTTAATGATCATTCTTTT
GTTAACATTACTGTCTCTGCTTCCTTTGGTGGTCATAGTGGTGCCAACCTTATTGCATCT
GACACTACTATCAATGGGTTTAGTTCTTTCTGTGTTGACACCAGACAATTTACCATTTCA
CTGTTTTATAACGTTACAAACATTTATGGTTATGTGTCTAAATCACAGGACAGTAATTGC
CCTTTCACCTTGCAATCTGTTAATGATTACCTGTCTTTTAGCAAATTTTGTGTTTCCACC
AGCCTTTTGGCTAGTGCCTGTACCATAGATCTTTTTGGTTACCCTGAGTTTGGTAGTGGT
GTTAAGTTTACGTCCCTTTACTTTCAATTCACAAAGGGTGAGTTGATTACTGGTACGCCT
AAACCACTTGAAGGTGTCACGGACGTTTCTTTTATGACTCTAGATGTGTGTACCAAGTA
TACTATCTATGGCTTTAAAGGTGAGGGTATCATTACCCTTACAAATTCTAGCTTTTTGGC
AGGTGTTTATTACACATCTGATTCTGGACAGTTGTTAGCCTTTAAGAATGTCACTAGTG
GTGCTGTTTATTCTGTTACGCCATGTTCTTTTTCAGAGCAGGCTGCATATGTTGATGATG
ATATAGTGGGTGTTATTTCTAGTTTGTCTAGCTCCACTTTTAACAGTACTAGGGAGTTGC
CTGGTTTCTTTTAC
